# Environmental Influences on the Spatial Ecology of Juvenile Smalltooth Sawfish (*Pristis pectinata*): Results from Acoustic Monitoring

**DOI:** 10.1371/journal.pone.0016918

**Published:** 2011-02-11

**Authors:** Colin A. Simpfendorfer, Beau G. Yeiser, Tonya R. Wiley, Gregg R. Poulakis, Philip W. Stevens, Michelle R. Heupel

**Affiliations:** 1 Center for Shark Research, Mote Marine Laboratory, Sarasota, Florida, United States of America; 2 Florida Fish and Wildlife Conservation Commission, Fish and Wildlife Research Institute, Charlotte Harbor Field Laboratory, Port Charlotte, Florida, United States of America; Smithsonian's National Zoological Park, United States of America

## Abstract

To aid recovery efforts of smalltooth sawfish (*Pristis pectinata*) populations in U.S. waters a research project was developed to assess how changes in environmental conditions within estuarine areas affected the presence, movements, and activity space of this endangered species. Forty juvenile *P. pectinata* were fitted with acoustic tags and monitored within the lower 27 km of the Caloosahatchee River estuary, Florida, between 2005 and 2007. Sawfish were monitored within the study site from 1 to 473 days, and the number of consecutive days present ranged from 1 to 125. Residency index values for individuals varied considerably, with annual means highest in 2005 (0.95) and lowest in 2007 (0.73) when several *P. pectinata* moved upriver beyond detection range during drier conditions. Mean daily activity space was 1.42 km of river distance. The distance between 30-minute centers of activity was typically <0.1 km, suggesting limited movement over short time scales. Salinity electivity analysis demonstrated an affinity for salinities between 18 and at least 24 psu, suggesting movements are likely made in part, to remain within this range. Thus, freshwater flow from Lake Okeechobee (and its effect on salinity) affects the location of individuals within the estuary, although it remains unclear whether or not these movements are threatening recovery.

## Introduction

Environmental influences on the spatial ecology of elasmobranchs have been poorly investigated. The effect of seasonal temperature changes on the broad-scale distribution of species has been widely reported [1], although the mechanisms and specific tolerances have rarely been investigated [2], [3]. Research has revealed that salinity plays an important role in the movement and distribution of nearshore and estuarine species [4]. Bull sharks (*Carcharhinus leucas*) are able to tolerate a wide range of salinities [5], but young juveniles have recently been shown to move so they remain at salinity levels between 7 psu and 20 psu [6], [7], [8]. Heithaus et al. [9] also demonstrated that for bull sharks in some estuarine habitats dissolved oxygen levels can influence movements and distribution more than salinity. Other nearshore species that have been shown to have movements affected by salinity include bonnetheads (*Sphyrna tiburo*) [10], sandbar sharks (*Carcharhinus plumbeus*) [11], and bat rays (*Myliobatis californica*) [12]. Given the importance of salinity, changes in freshwater flow regimes into estuaries as a result of climate change or water management practices will affect populations by potentially changing their distributions.

The sawfishes (Family Pristidae) were once common inhabitants of tropical and subtropical inshore, estuarine, and freshwater areas world-wide [13]. However, pressure from fishing and habitat loss have led to population declines [14] and all species are currently Critically Endangered on the IUCN Red List (see www.redlist.org); and some species are protected under national endangered species legislation. The smalltooth sawfish (*Pristis pectinata*) was listed as Endangered by the United States National Marine Fisheries Service (NMFS) and protected by the Endangered Species Act in 2003. Although once prevalent throughout Florida and commonly encountered from Texas to North Carolina, *P. pectinata* currently occurs mostly in south and southwest Florida [15], [16], [17]. It grows to over 500 cm STL (stretched total length) after being born in estuarine and nearshore areas at sizes between 69 and 81 cm STL [18]. One of the objectives of the recovery plan for *P. pectinata* is to protect or restore habitats for the juveniles that occur in estuarine and nearshore areas [19]. However, little is known about their long-term habitat use and movements, or how environmental factors affect these attributes, as large-scale spatial studies of *P. pectinata* ecology are lacking.

Studies of the movements of sawfish are limited. Thorson [5] reported tag and recapture results of largetooth sawfish (*Pristis perotteti*) showing that they moved between freshwater and saltwater in Nicaragua. Based on acoustic monitoring results, the freshwater sawfish (*P. microdon*), which spends the first few years of life in rivers [20], has been shown to ontogenetically partition depth within a river system in northern Australia [21]. Simpfendorfer et al. [22] used acoustic tracking and acoustic monitoring to investigate the short-term movements, site fidelity, and habitat use of juvenile *P. pectinata* in southern Florida. These studies have shown that for juvenile sawfish, very shallow depths are a critical factor, probably because of the protection that it can provide from predators, and that there are clear ontogenetic changes in habitat use. To date, there is no published information on how environmental factors can influence movement and distribution of *P. pectinata*. However, such information may be critical to recovery of this population as they occur adjacent to the Florida Everglades which is undergoing major restoration that is significantly altering freshwater flow patterns in southern Florida (see www.evergladesplan.org).

To investigate the role that changes in environmental conditions have on the movement and distribution of *P. pectinata*, an acoustic array was deployed within the estuarine portion of the Caloosahatchee River in southwest Florida to track their long-term movements. The specific aims of this study were to determine the level of residency, movement patterns and activity space within the system, and investigate how these attributes were influenced by ontogeny and changes in environmental conditions.

## Methods

### Ethics statement

This research was conducted in accordance with National Marine Fisheries Service Endangered Species Permit numbers 1352 and 1475. Animal ethics approval was granted by Mote Marine Laboratory to Colin Simpfendorfer.

### Study site

**Figure 1:**
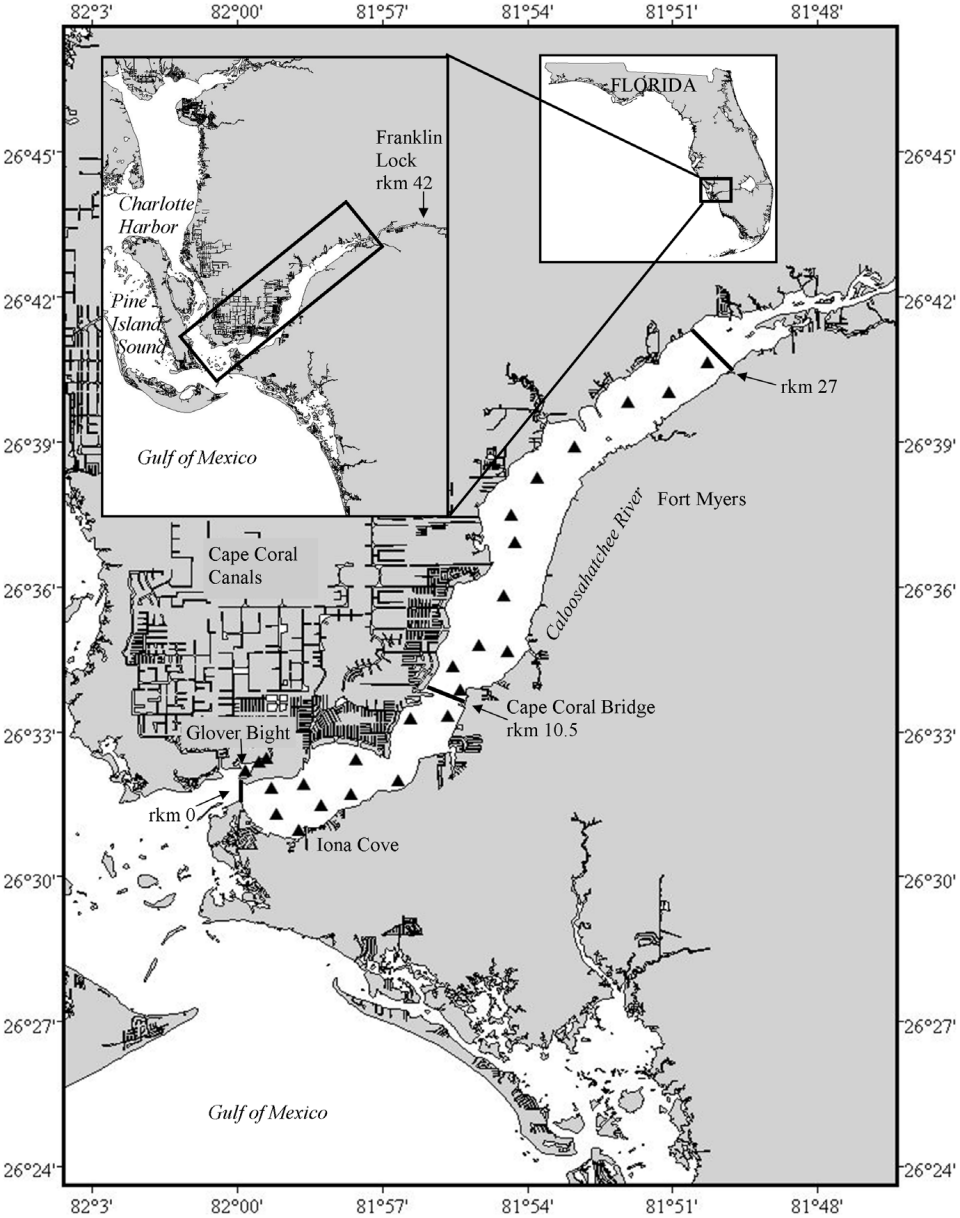
Study location in the Caloosahatchee River indicating locations of acoustic receiver stations (▴) within the river. Insets show study site location on the central Gulf of Mexico coast of Florida. rkm, river kilometer.

**Figure 2:**
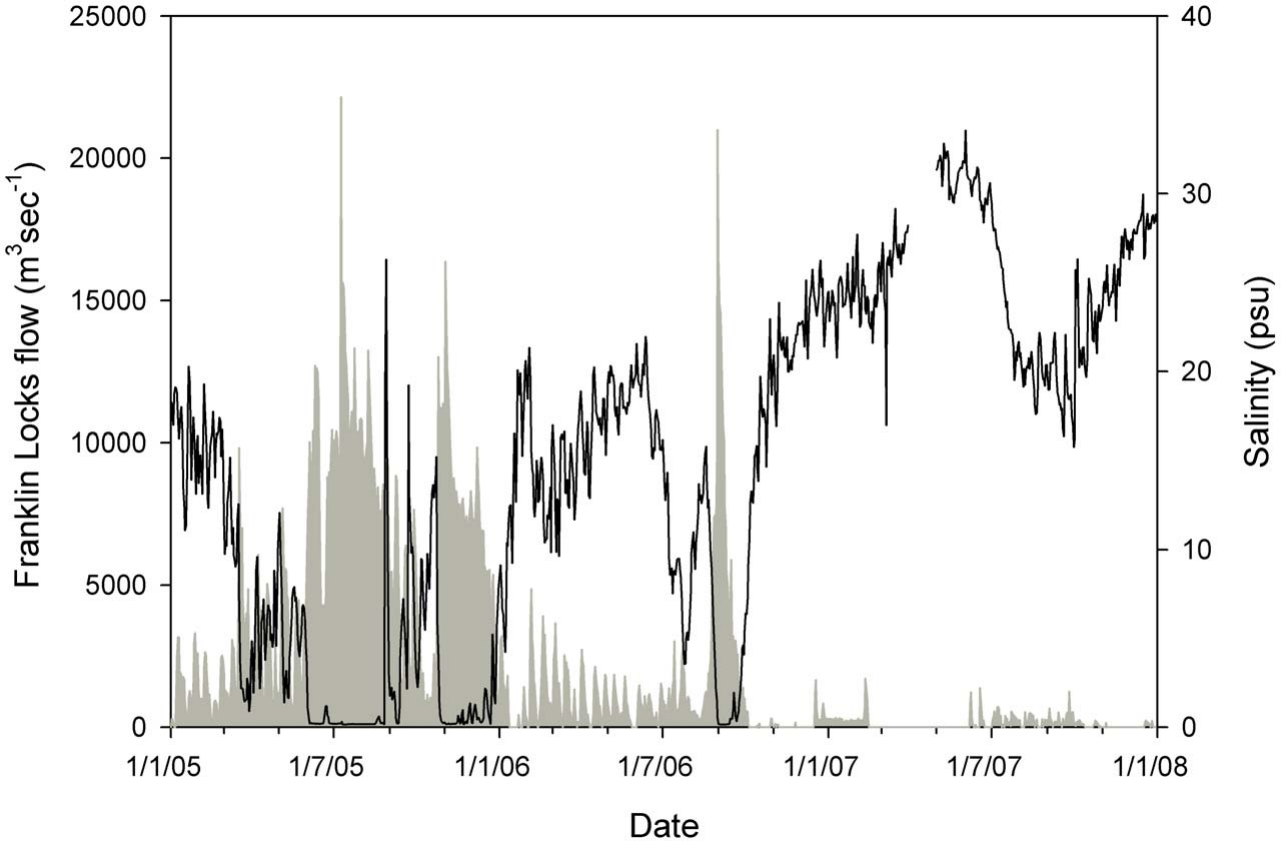
Environmental conditions within the study area. 2005 was a wet year with high freshwater flows through the Franklin Locks (grey shaded area) of long periods with salinity at Cape Coral (black line) below 10 psu. 2006 was a moderately wet year with a short period of high flow, and 2007 was a dry year with very small flows and high salinity.

This study was conducted in the lower 27 km estuarine portion of the Caloosahatchee River in southwest Florida (Figure 1). The river connects Lake Okeechobee to the Gulf of Mexico and is the major source of freshwater to southern Charlotte Harbor. Water from Lake Okeechobee also flows to the east coast via the St Lucie River, and changes in the distribution between the two systems over time has affected the levels of freshwater flow. Freshwater flows in the Calooshatchee River during the current study were greatest during summer (Figure 2) and varied considerably between years depending on the magnitude of the wet season. The river has been substantially altered in the last 100 years [23], including an artificial link to Lake Okeechobee, extensive canal systems, three locks to permit boat passage, and dams to regulate water flow. The upper reaches of the study site had natural shoreline and native vegetation (primarily red mangroves *Rhizophora mangle*) while closer to the mouth the habitat was largely altered by urbanization including extensive canal developments and shoreline modification associated with the cities of Cape Coral and Fort Myers.

### Field methods

A series of 25 VR2 acoustic receivers (Vemco Ltd.) were deployed within the study site to passively track the movements of *P. pectinata* fitted with acoustic tags (Figure 1). Methods for deploying receivers have previously been described [24], [25]. Acoustic receivers were deployed in August 2003 and were continuously present within the study site until project completion in October 2007. These single frequency, omnidirectional data logging receivers recorded the time, date, and identity of *P. pectinata* fitted with acoustic transmitters that swam within the detection range. Receivers had a maximum detection range of approximately 800 m [26]. Detection range within the Caloosahatchee River allowed *P. pectinata* to often be detected on more than one receiver simultaneously and the receiver array allowed individuals to be continuously monitored while they were present within the study area. Approximately once per month data were downloaded from receivers and any required maintenance (e.g. removal of biofouling organisms and battery change) was performed.

Sampling for the capture of *P. pectinata* was conducted with longlines, gill nets, seine nets, and rod and reel (detailed field methods can be found in Simpfendorfer et al. [18]). All captured individuals were measured (stretch total length), sexed and fitted with an external identification tag. *Pristis pectinata* to be tracked were fitted with Vemco RCODE V9, V13 or V16 individually coded transmitters mounted on Rototags or Jumbo Rototags (Dalton, UK) and attached to the first or second dorsal fin. External attachment was required due to Endangered Species Act permitting limitations. Each transmitter was coded with a unique pulse series and operated on 69.0 kHz at random intervals between 60 and 180 seconds. Random signal transmission times prevented more than one signal continuously overlapping and blocking detection by a receiver. Expected battery life of transmitters was approximately 8 months (V9, V13) or 18 months (V16).

### Data analysis

Occurrence of tagged *P. pectinata* in the array was determined on a daily basis and a presence history was plotted to provide a visually interpretable timeline of occurrence throughout the study period. The number of days individuals were detected on receivers in the study area, the total number of days from the first to last detection, and the maximum number of consecutive days present were calculated. A residency index was calculated as the ratio between the number of days an animal was detected to the number of days from the first to the last detection, with a value of one indicating it was detected every day and zero indicating it was never detected. Residency index values were compared between years with size and the total number of days monitored as covariates using analysis of covariance (ANCOVA). A post-hoc Tukeys unequal N Honest Significant Difference (HSD) test was used to determine years that were significantly different from each other.

The location of each tagged *P. pectinata* in the estuary was estimated every 30 minutes using the receiver distance algorithm described by Simpfendorfer *et al.* [24]. This algorithm used data from the receiver array to estimate the distance between the center of activity (COA) of each individual *P. pectinata* and the river mouth (river distance in river kilometers [rkm] = 0 km). The COA positions were used to generate daily minimum and maximum river distances, and mean daily river distances for individual *P. pectinata*. Daily, weekly, and monthly activity spaces were calculated as the difference between the maximum and minimum river distance for the relevant time period [26], [27]. Analysis of covariance was used to test for differences in daily activity space between sexes and length (covariate). The pattern of movement of individuals between COA positions was assessed using a General Linear Model (GLM) with diel period (day or night), month, river distance (covariate) and the interaction between diel period and month.

To investigate how environmental factors influenced the distribution of *P. pectinata* within the estuary the daily mean river distance of each individual was regressed separately against temperature, salinity and freshwater inflow. Where necessary, mean river distance was log-transformed to meet conditions of normality. Daily mean values of salinity and temperature data were obtained from the South Florida Water Management District (SFWMD) Cape Coral Bridge station, approximately 10.5 km from the river mouth (in the middle of the study area). The salinity and temperature at the Cape Coral Bridge was used as an index of the salinity regime present in the river on each day. Daily freshwater inflow to the estuary was obtained from the SFWMD recording station at the Franklin Locks upstream of the study area (river distance = 42 km). Salinity was significantly negatively correlated with freshwater inflow (R^2^ = 0.591, p<0.0001), but also depended on rainfall [6]. The number of *P. pectinata* present each day in the main stem of the river was calculated and compared to the daily freshwater inflow through the Franklin Lock and salinity measured at the Cape Coral Bridge.

Electivity analysis was used to determine if *P. pectinata* exhibited affinity for, or avoidance of, specific salinity conditions within the river. To do this, the salinity in which individuals occurred each day was compared to those available in the river using Chesson's α [28]:
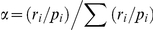
where *r_i_* is the proportion of time an individual spent in salinity *i* and *p_i_* is the proportion of salinity *i* available in the river. Since different years had different salinity regimes and not all salinities were available in each year, annual electivity values were standardised using the method described by Heupel and Simpfendorfer [6]. The salinity for a specific day and river distance (*s_t,i_*) for any given location within the river (*i*) was estimated using the equation from Heupel and Simpfendorfer [6]:
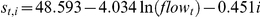
where *flow_t_* is the freshwater inflow rate for day *t* into the estuary at the Franklin Lock. River distances for the electivity analysis were taken as the daily mean river distance of each individual.

To investigate the combined effects of environmental and other factors on the distribution of juvenile *P. pectinata* within the Caloosahatchee River estuary a Generalized Additive Model (GAM) was used to model the distribution of individuals on a daily basis within the river based on five factors: salinity, temperature, freshwater inflow, month and sawfish length. All factors were continuous except for month. Sawfish lengths of <100 cm corresponded to neonate individuals, those 100–140 cm were up to one year old, those 141–180 cm were one to two years old and those >180 cm were older than two years [18]. Twenty-five different models were constructed ranging from simple single factor models to multifactor models with interaction terms. The model fit was determined using the Aikake Information Criteria (AIC) and the factors of the model with the lowest AIC value and highest AIC weight were considered to be those that best explained sawfish distribution within the river.

## Results

**Figure 3:**
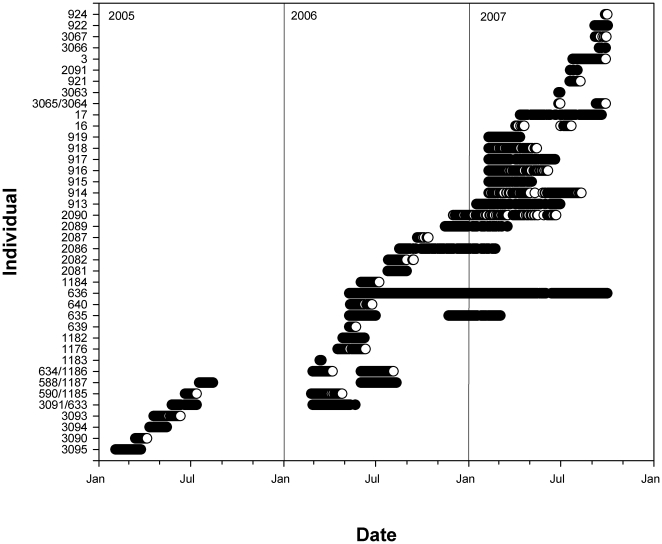
Presence history of the monitored *Pristis pectinata* in the Caloosahatchee River from 2005 to 2007.

Forty juvenile *P. pectinata* were tagged for monitoring in the Caloosahatchee River between 2005 and 2007 (Figure 3). Individuals ranged in size from 69 to 250 cm STL (mean = 149 cm STL) representing neonate, young-of-the-year, and juveniles (Table S1). Captures occurred in all months except October. Five individuals were recaptured during the study and fitted with a transmitter a second time. The total monitoring period from first to last detection for individuals ranged from 3 to 510 d, individuals were detected within the monitoring area from 2 to 473 d and maximum consecutive periods present ranged from 1 to 125 d.

Residency index values of individuals ranged from 0.23 to 1.0. Annual mean residency index values were significantly different between years (ANCOVA, F_2,35_ = 4.65, p = 0.016), with values highest in 2005 (0.95), moderate in 2006 (0.83) and lowest in 2007 (0.72). Residency index values were significantly related to the numbers of days present (ANCOVA, F_1,35_ = 4.42, p = 0.043), but not size (ANCOVA, F_1,35_ = 1.95, p = 0.171). Post-hoc tests showed that 2005 and 2007 were significantly different from each other, while 2006 was not significantly different from either 2005 or 2007. The lower value of residency index in 2007 was in part related to some tagged sawfish moving upstream out of the monitoring array. This occurrence was evidenced by four individuals (915, 918, 2089, 2090) being detected on equipment maintained by the Florida Fish and Wildlife Conservation Commission (FWC) upstream from the study area for 2 to 55 d between March and June of 2007 (R. Taylor, unpublished data). Thus, these individuals were still in the river, but outside of the study area and not considered in the residency index.

**Figure 4:**
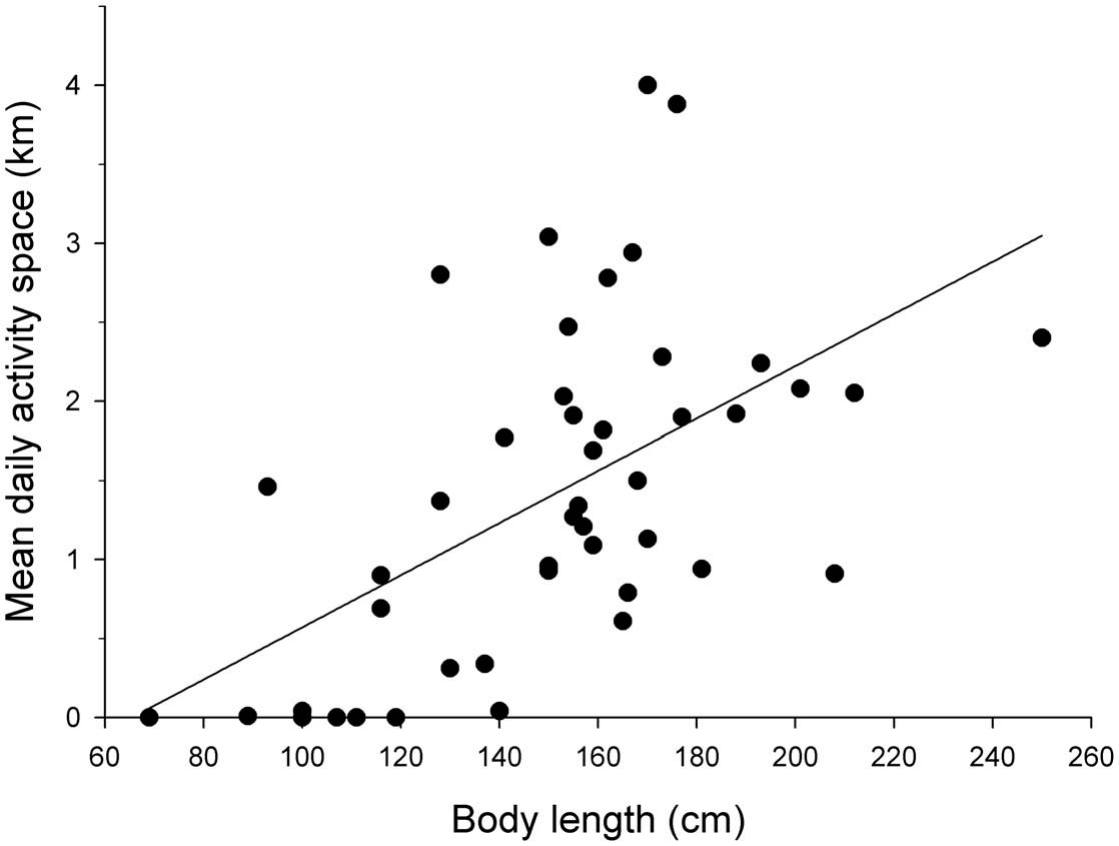
Relationship between body length and mean daily activity space of *Pristis pectinata* monitored in the Caloosahatchee River.

Individual *P. pectinata* had relatively small daily activity spaces, but covered the entire study area over the long-term. Mean daily activity spaces ranged from 0.0 km (indicating that an individual was heard on only a single receiver for the whole day) to 3.88 km (Table 1); the mean across all individuals was 1.42 km. Individual daily activity space values were mostly less than 5 km, with estimates of less than 1 km common (2005: 44.9%; 2006: 54.3%; 2007: 56.3%). On rare occasions, values >10 km were observed. Mean daily activity space was not significantly different between males and females (ANCOVA, F_1,40_ = 0.107, p = 0.745) but was significantly related to individual size (ANCOVA F_1,40_ = 14.0, p<0.001 ), with larger individuals having larger activity space (Figure 4).

**Table 1:** Comparison of General Additive Models constructed for predicting juvenile *Pristis pectinata* locations within the Caloosahatchee River (as defined by the distance from the river mouth (rkm)) in southwest Florida.

Index	Model	AIC	AIC weight
1	rkm∼1	16944	<0.0001
2	rkm∼s(sal)	13588	<0.0001
3	rkm∼s(temp)	16566	<0.0001
4	rkm∼s(len)	16406	<0.0001
5	rkm∼s(mon)	16611	<0.0001
6	rkm∼(ln(flow))	16281	<0.0001
7	rkm∼s(sal)+s(temp)	13448	<0.0001
8	rkm∼s(sal)+s(ln(flow))	13585	<0.0001
9	rkm∼s(sal)+s(len)	13004	<0.0001
10	rkm∼s(sal)+s(mon)	13327	<0.0001
11	rkm∼s(sal)+s(ln(flow))	12995	<0.0001
12	rkm∼s(sal)+s(ln(flow))+s(mon)	13326	<0.0001
13	rkm∼s(mon)+s(ln(flow)+s(len)	15376	<0.0001
14	rkm∼s(sal)+s(ln(flow))+s(len)+s(mon)	12800	<0.0001
15	rkm∼s(sal)+s(ln(flow))+s(sal,ln(flow))	13524	<0.0001
16	rkm∼s(sal)+s(temp)+s(sal,temp)	13414	<0.0001
17	rkm∼s(sal)+s(len)+s(sal,len)	12387	<0.0001
18	rkm∼s(sal)+s(mon)+s(sal,mon)	13267	<0.0001
19	rkm∼s(sal)+s(temp)+s(sal,temp)	12648	<0.0001
20	rkm∼s(sal)+s(len)+s(sal,len)+s(mon)	12180	<0.0001
21	rkm∼s(sal)+s(len)+s(sal, len)+s(sal, temp)	11997	<0.0001
22	rkm∼s(sal)+s(len)+s(sal,len)+s(len,mon)	11865	>0.9999
23	rkm∼s(sal)+s(len)+s(sal,temp)+s(len,mon)	12308	<0.0001
24	rkm∼s(sal)+s(len)+s(mon)+s(sal,temp)	12543	<0.0001
25	rkm∼s(sal)+s(len)+s(mon)+s(sal,temp)+s(len,mon)	12212	<0.0001

Models incorporate salinity (sal), water temperature (temp), length (len), month (mon) and freshwater flow (flow). Interaction terms are indicated by two factors within a term. Models with the lowest AIC value indicate the most plausible model, in this case number 22. AIC weight indicates the proportional support for the individual models.

Mean weekly activity space of individual *P. pectinata* were larger than mean daily activity space (range 0–10.1 km), often by a factor of two or more. This suggests that individuals moved along the river over periods of several days rather than staying in the same location over longer periods. Monthly mean activity space of individuals was greater than mean weekly values, but by less than a factor of two (Table 1), suggesting that activity space was relatively stable over time frames >7 d. The largest individual monthly activity space estimates in 2006 and 2007 were 22.5 and 23.8 km, respectively, suggesting occasional use of nearly the entire study area within a month.

The majority of movements between 30 minute COA positions (91%) were less than 1.0 km, indicating that longer distance movements were rare. Movements between COA positions were significantly related to month (GLM, F_9,18800_ = 2.06, p = 0.029) and river distance (GLM, F_1,18800_ = 86.4, p<0.001). Individuals that were located upstream were more likely to move downstream than those located downstream. There was no significant diel difference in the movement of individual *P. pectinata* between COA positions (GLM, F_1,18800_ = 0.25, p = 0.62), with overall movements of equal magnitude likely up or down the river either day or night. However, there was a significant interaction between diel period and month (GLM, F_9,18800_ = 2.45, p<0.001). This interaction suggests that there were monthly differences in the diel movement patterns of *P. pectinata*. Large movements between COA positions (>5 km) occurred very rarely, mostly in 2007, but were not associated with major freshwater flow events.

**Figure 5:**
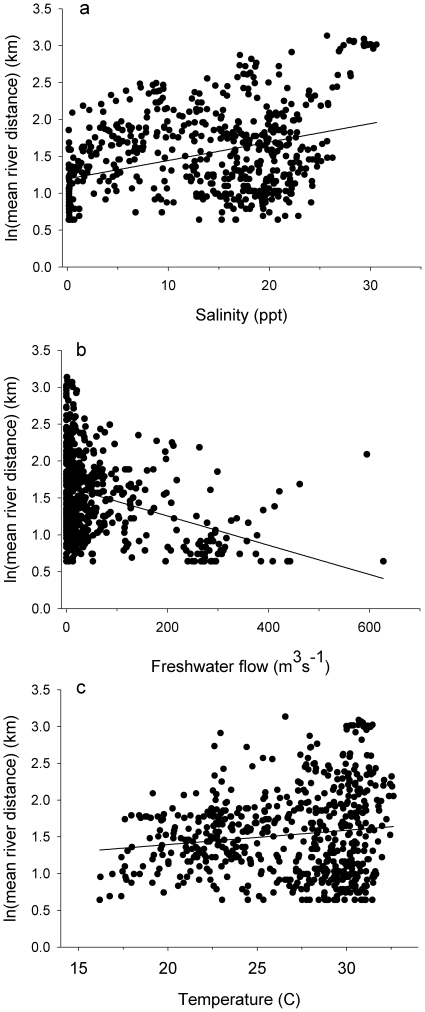
Relationship between distribution of tagged sawfish and environmental parameters. (a) salinity, (b) freshwater flow and (c) temperature.

**Figure 6:**
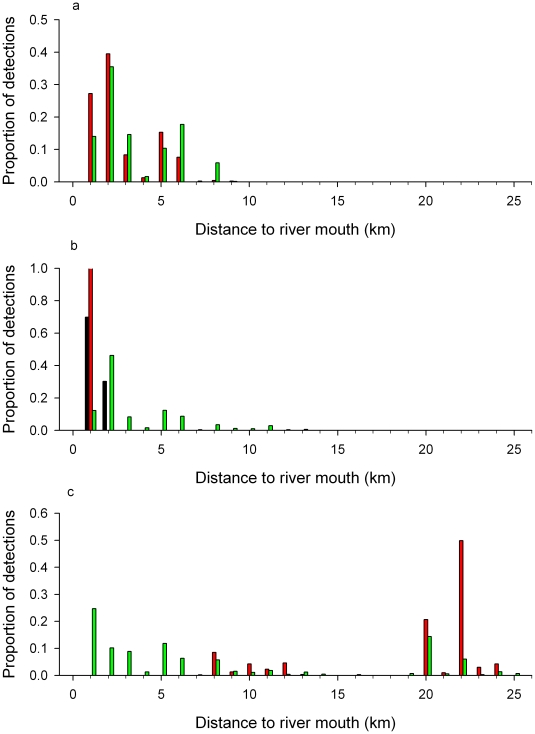
Distribution of neonate (black bars), young-of-the-year (red bars) and >1 year old (green bars) *Pristis pectinata* within the Caloosahatchee River. (a) 2005, (b) 2006 and (c) 2007 based on detections by acoustic receivers. Location was calculated as kilometers from the river mouth using a linear mean-position algorithm.

**Figure 7:**
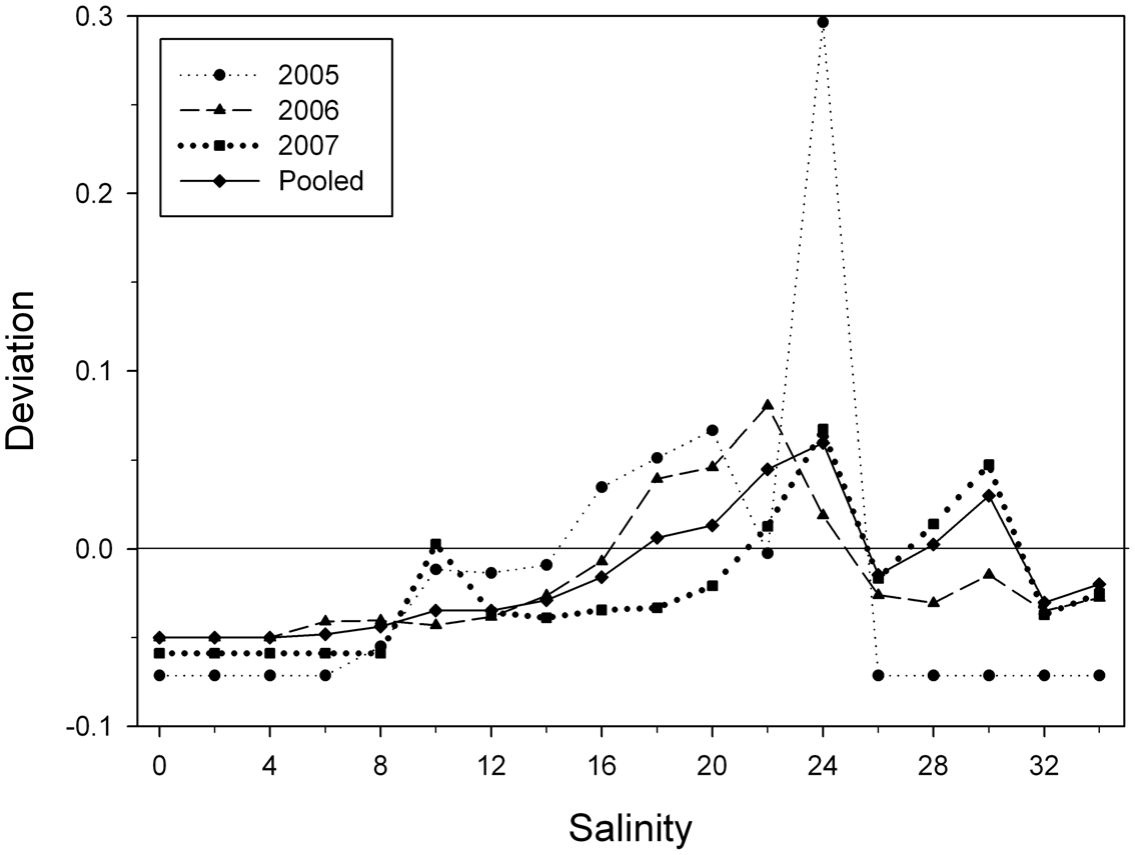
Salinity electivity of *Pristis pectinata* in the Caloosahatchee River estuary revealing a preference for salinities from 18 to at least 24 psu. Sample sizes above 24 psu were small, limiting the ability to make conclusions.

During the study, water temperature ranged from 14.6 to 32.6 C, salinity ranged from 0.1 to 33.6 psu and freshwater inflow ranged from 0.0 to 627.4 m^3^s^−1^. *Pristis pectinata* were present throughout the entire range of these environmental conditions. Salinity was negatively correlated with flow (r^2^ = 0.646, p<0.001). There were positive correlations between *P. pectinata* log-transformed mean daily river location and salinity (Figure 5a; r^2^ = 0.126, p<0.001) and log-transformed mean daily river location and temperature (Figure 5c; r^2^ = 0.016, p<0.05). There was a negative relationship between log-transformed mean river location and flow (Figure 5b; r^2^ = 0.116, p<0.001). Distribution of *P. pectinata* within the river (Figure 6) indicated a significantly different proportion of detections by river location between years (χ^2^ = 28766, df = 48, p<0.001). Differences between years were likely driven in part by differences in flow regime. During 2005, when flows were high, individuals were in the lower reaches of the river, while periods of little or no flow in 2007 corresponded to periods when individuals were far upriver. This suggests flow, in conjunction with physical factors such as depth, plays some role in individual location within the river, possibly through their influence on salinity. Electivity analysis demonstrated that *P. pectinata* had an affinity for salinity values between 18 and at least 24 psu (Figure 7). At salinities above 24 psu sample sizes were small and conclusions limited.

**Figure 8:**
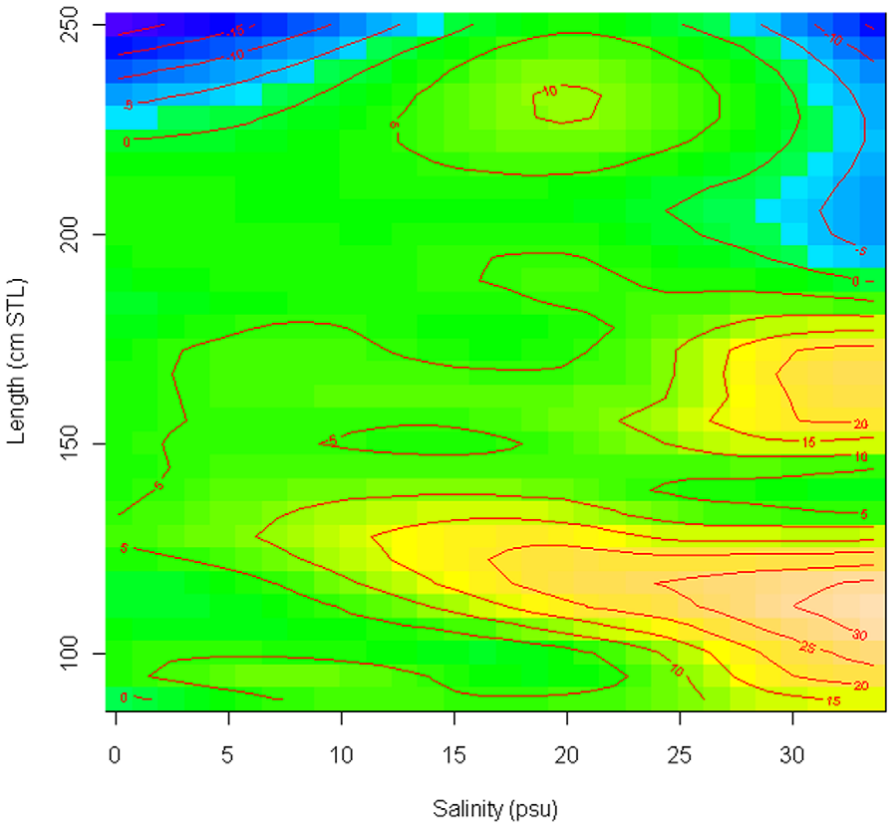
Predictions of river distance (contour lines, colors) based on salinity and sawfish length from the best fitting Generalized Additive Model for *Pristis pectinata* in the Caloosahatchee River. Blues indicate areas downstream of the study area, green indicates low reaches of the study area, yellow mid-reaches and white upper reaches.

The best fitting GAM model included the factors salinity, length, and the interactions salinity*length, and month*length (Table 1). This model demonstrated that as salinity increased sawfish moved upriver, but that the salinities that different size classes moved at were different (Figure 8). The model predicted that neonate individuals (<100 cm) had limited movements in relation to salinity, while individuals between 100 cm and 140 cm (up to ∼1 year old) moved further upriver and started moving at the lowest salinities. Individuals from 141 cm to 180 cm (1–2 years old) were predicted to move upriver at much higher salinities (i.e., they may be more tolerant of salinity changes), and not move as far as the 100 cm to 140 cm size class. Individuals >180 cm were predicted to have limited movements in relation to salinity, although sample sizes were relatively small. The interaction between month and length probably occurred because of the rapid growth of this species [18] that meant particular size classes were only available in particular months, and so accounting for this through the interaction increased the fit of the model.

## Discussion

The results from acoustic monitoring within the Caloosahatchee River show that juvenile *P. pectinata* are often present within estuarine areas for at least the first two years of life. This observation is consistent with results from short-term acoustic tracking [22] and encounter data [17] that indicate that individuals move away from shallow inshore habitats to deeper areas at sizes >250 cm (∼2 yr old). Presence histories of individuals were mostly relatively short (∼3 months), but this did not necessarily reflect animals moving out of the Caloosahatchee River estuarine system. Rather, the requirements to deploy acoustic tags externally to meet permitting restrictions meant that many were often prematurely shed due to rapid growth rates of juvenile *P. pectinata* [18]. Juveniles are therefore likely to remain within the Caloosahatchee River estuary for most or all of the first few years of life (as suggested by high mean residency values), possibly only leaving when environmental conditions are less favorable. This observation is similar to that of Thorburn et al. [20] who reported that freshwater sawfish *Pristis microdon* remained within the Fitzroy River in northern Australia for most of its juvenile life. However, *P. pectinata* does not appear to remain in the estuary until it matures, instead leaving after approximately two years, before the size at which they mature. The bull shark, which also uses the Caloosahatchee River estuary as a nursery area followed a similar pattern, leaving after about two years [6], [7], well before they matured.

Within the estuary several factors influenced where *P. pectinata* occurred. The two most important factors were salinity regime and size. Changes in salinity could directly affect sawfish physiology or could be a proxy for indirect effects such as prey distribution that determine sawfish location in the river. During high freshwater flow conditions in 2005 and 2006, sawfish of all sizes remained in the lower portion of the river. Increases in salinity (measured at the mid-point of the study area) as a result of reductions in freshwater flow, likely caused individuals to move upriver in 2007 to meet their salinity requirements as drier conditions prevailed in the study area. Similar salinity effects have been observed in several other elasmobranch species that occur in estuarine and nearshore habitats [6], [8], [26], [29]. However, for *P. pectinata* the response to changes in salinity regime differed among individuals of different ages. Individuals approaching 1 year of age (100–140cm) were most mobile, moving up river at the lowest salinity levels. The exact reasons for this sensitivity is unclear, but may relate to the energetic costs of osmoregulation [6], or the movement of preferred prey in response to salinity changes [8]. The youngest individuals (i.e., neonates) did not demonstrate this level of sensitivity despite a smaller body size which increases the cost of osmoregulation due to higher surface area to volume ratio. However, these animals are more vulnerable to predation due to their small size, and fine-scale acoustic tracking has demonstrated that this size class has small activity spaces in very shallow habitats, probably to avoid predation [22]. Thus, the energetic cost of osmoregulation for this size class may be outweighed by the increase in survival that results from remaining in these protected habitats. Behavioral choices that lead to higher energetic costs are commonly observed in aquatic organisms [30] demonstrating the importance of predation risk in determining the movements and distribution of some species. Tracking data [22] also indicated that *P. pectinata* transition from restricted activity space to more broad use of space at ∼100 cm, a result consistent with the predictions of the GAM for when individuals become most sensitive to changes in salinity regime. Tracking data indicate that this is a transition from remaining in very shallow areas, often mud banks in mangrove areas, to following mangrove shorelines. Individuals >1 yr of age may be less sensitive to salinity changes because of their larger body size, and so only respond to changes when freshwater flows have substantially decreased.

Though not the sole cause of sawfish movements, the influence of salinity was further supported by the results of electivity analysis, which demonstrated an affinity for areas with salinities between 18 and at least 24 psu. The movements of *P. pectinata* in response to changes in salinity may thus reflect individuals seeking to remain within this salinity range. As freshwater flows into the estuary decrease, the location of saltier water also moves further upriver as the marine influence increases through tidal movement. Research on bull sharks within the same system determined that they had an affinity for salinities between 7 and 20 psu [6], [7]. This difference in salinity range may reduce predation on juvenile *P. pectinata* by facilitating a separation between these two species. While there is no direct evidence for bull sharks consuming *P. pectinata*, predation has been observed on other species of *Pristis* [31]. An affinity for lower salinities than most marine predators also means that risk from that large potential source of predation is also lowered. Thus, juvenile *P. pectinata* appear to have multiple approaches to reducing predation risk.

Daily activity spaces of *P. pectinata* were relatively small compared to other species within the same system. Collins et al. [32] reported that cownose rays had an overall mean daily activity space of ∼3.5 km of river, while Heupel et al. [27] reported daily activity spaces values of ∼4 km for bull sharks. These values were >2 times larger than that for *P. pectinata*. This difference is attributed to disparate activity levels between these species. Both bull sharks and cownose rays are semi-pelagic species swimming almost continuously [8], [26], while *P. pectinata* is a benthic species that spends considerable periods of time (∼21% of time) resting on the bottom [22]. This conclusion is also supported by the observation in the current study that there was no movement between >90% of consecutive COA positions. Direct comparison to other studies on elasmobranchs, however, were inappropriate because most studies normally use area measurements [1] not linear measures as is often used in river studies. The observation of a size effect on activity space was consistent with results from active tracking, which demonstrated an increase in home range and rate of movement with size [22].

The movements between COA positions demonstrated that the short-term movement patterns observed using active tracking [22] occur through the juvenile life stages and were not an artifact of capture affecting behavior. The difference in movement direction between upriver and downriver locations was likely related to behavior that enabled individuals to locate or remain in their preferred salinity. Behavioral mechanisms by which marine animals maintain the presence in locations with preferred attributes has been poorly studied in elasmobranchs. The use of behavior to achieve homeostasis has been suggested for both temperature [33], [34] and salinity [6], but the mechanisms used to achieve it remain unknown [4]. The highly seasonal nature of rainfall (and hence freshwater inflow) in southern Florida was the most likely driver of the differences in movement patterns by month, with tendencies to move down river during wetter months during the summer and upriver during drier months, especially in autumn. Overall, the movements of *P. pectinata* were consistent with those of a species that had an affinity for a particular salinity range.

The results of this study demonstrate that water management practices will have effects on *P. pectinata*. The preference of juveniles for salinities between 18 and at least 24 psu means that as freshwater flows into systems decrease, they are likely to move higher into estuaries. The extent of these movements is related to the magnitude of salinity change. When flow patterns are changed, individuals may move to areas with their preferred salinity, but habitats within these areas may be less (or more) suitable than those previously occupied [35]. Within the Caloosahatchee River, increases in salinity that led to *P. pectinata* occurring upriver of the study area may be most problematic as the river becomes quite narrow with few shallow habitats that this species appears to use as a refuge from predation [22]. In addition, water management decisions related to the amount of flow from Lake Okeechobee via the Calooshatchee and St Lucie rivers may have important implications for conservation measures.

More broadly, flow regimes within any estuaries where *P. pectinata* occurs that result in animals being distributed in sub-optimal habitats may reduce survival and thus hinder the recovery of this population. Similarly, water management practices that result in repeated large changes in flow over short periods of time will result in large amounts of movement between different habitats which will increase energy expenditure, and may expose individuals to greater risks of predation. The most vulnerable portion of the population to effects from water management practices appear to be sawfish in their first year of life. Neonate animals remain in small patches of shallow habitat irrespective of salinity, suggesting that they may suffer greater osmotic stress if salinity within these areas falls outside their preferred range for long periods. Although neonate sawfish captured downriver during high flow periods exhibit fast growth [18], growth and survivorship of neonates located further upriver during drought conditions is unknown. Water management practices therefore need to be considered in relation to the recovery of the *P. pectinata* population. More information on the energetic costs associated with occupying different salinities and the effects of occupying sub-optimal habitats on survival of juvenile *P. pectinata* must be determined.

The results of this and similar studies [6], [7], [9], [27], [32] have demonstrated that water management practices can have significant effects on elasmobranchs that inhabit estuaries. Ensuring that water flows are managed to meet the physiological and ecological needs of these important species, especially those like *P. pectinata* that face conservation challenges, will ensure healthy estuarine ecosystems. Research that continues to increase the understanding of how environmental factors influence the movements and distribution of estuarine elasmobranchs will be required to enable water managers to effectively implement flow regimes that meet these needs.

## Supporting Information

Table S1 **Presence and activity space data for** ***Pristis pectinata*** **monitored in the Caloosahatchee River from 2005 to 2007.** Transmitter numbers with identical numbered superscripts indicate individuals that were recaptured and fitted with an additional transmitter at a later date. Size, detection and activity space data reflect the two periods of monitoring for these individuals. STL, stretch total length; t_det_, number of days detected; t_max_, number of days from first to last detection; t_con_, maximum number of consecutive days present; RI, residence index; AS_d_, mean daily activity space; AS_w_, mean weekly activity space; AS_m_, mean monthly activity space.
(DOC)
