# Supplementary material for: Environmental Influences on the Spatial Ecology of Juvenile Smalltooth Sawfish (Pristis pectinata): Results from Acoustic Monitoring
Source: PLoS One. 2011 Feb 11;6(2):e16918. doi: 10.1371/journal.pone.0016918 (PMC3037940; doi:10.1371/journal.pone.0016918)
Supplement: Table S1 — Presence and activity space data for Pristis pectinata monitored in the Caloosahatchee River from 2005 to 2007. Transmitter numbers with identical numbered superscripts indicate individuals that were recaptured and fitted with an additional transmitter at a later date. Size, detection and activity space data reflect the two periods of monitoring for these individuals. STL, stretch total length; tdet, number of days detected; tmax, number of days from first to last detection; tcon, maximum number of consecutive days present; RI, residence index; ASd, mean daily activity space; ASw, mean weekly activity space; ASm, mean monthly activity space. (DOC) [file pone.0016918.s001.doc]

Table S1. Presence and activity space data for *Pristis pectinata* monitored in the Caloosahatchee River from 2005 to 2007.

| Tag | Sex | STL  (cm) | t_det_  (d) | t_max_  (d) | t_con_  (d) | RI | AS_d_  (km) | AS_w_  (km) | AS_m_  (km) |
| --- | --- | --- | --- | --- | --- | --- | --- | --- | --- |
| 3095 | M | 141 | 48 | 51 | 19 | 0.94 | 1.77 | 3.94 | 4.5 |
| 3090 | F | 150 | 24 | 24 | 24 | 1.0 | 3.04 | 5.70 | 8.3 |
| 3094 | M | 173 | 35 | 35 | 35 | 1.0 | 2.28 | 4.29 | 5.77 |
| 3093 | M | 159 | 49 | 54 | 31 | 0.91 | 1.69 | 3.92 | 5.31 |
| 3091^1^  633^1^ | F  F | 181  208 | 42  75 | 50  85 | 25  68 | 0.84  0.88 | 0.94  0.91 | 1.47  1.71 | 2.0  1.80 |
| 590^2^  1185^2^ | F  F | 107  162 | 23  56 | 24  63 | 18  40 | 0.96  0.89 | 0.0  2.78 | 0.0  8.72 | 0.0  11.8 |
| 588^3^  1187^3^ | F  F | 116  177 | 27  71 | 27  71 | 27  71 | 1.0  1.0 | 0.90  1.90 | 1.53  4.88 | 2.10  8.17 |
| 634^4^  1186^4^ | F  F | 155  176 | 40  65 | 40  65 | 40  65 | 1.0  1.0 | 1.91  3.88 | 4.02  6.94 | 3.46  8.83 |
| 1183 | F | 69 | 4 | 4 | 4 | 1.0 | 0.0 | - | - |
| 1176 | M | 150 | 47 | 56 | 14 | 0.84 | 0.93 | 2.79 | 6.73 |
| 1182 | M | 165 | 44 | 45 | 44 | 0.98 | 0.61 | 1.63 | 3.13 |
| 639 | F | 89 | 12 | 14 | 8 | 0.86 | 0.02 | 0.03 | 0.1 |
| 640 | F | 166 | 37 | 44 | 14 | 0.84 | 0.79 | 2.5 | 4.7 |
| 635 | F | 100 | 140 | 299 | 36 | 0.47 | 0.04 | 0.06 | 0.06 |
| 636 | M | 93 | 473 | 510 | 125 | 0.93 | 1.46 | 4.28 | 7.30 |
| 1184 | F | 212 | 37 | 37 | 37 | 1.0 | 2.05 | 3.58 | 4.15 |
| 2081 | M | 188 | 37 | 38 | 22 | 0.97 | 1.92 | 3.58 | 4.5 |
| 2082 | M | 193 | 38 | 52 | 22 | 0.73 | 2.24 | 4.91 | 5.83 |
| 2086 | F | 128 | 108 | 190 | 13 | 0.57 | 2.80 | 7.71 | 15.71 |
| 2087 | F | 140 | 17 | 23 | 12 | 0.74 | 0.04 | 0.04 | 0.05 |
| 2089 | F | 167 | 70 | 124 | 21 | 0.56 | 2.94 | 10.09 | 13.62 |
| 2090 | M | 154 | 118 | 204 | 18 | 0.58 | 2.47 | 8.32 | 12.61 |
| 913 | M | 155 | 93 | 166 | 17 | 0.56 | 1.27 | 3.46 | 5.38 |
| 914 | F | 156 | 119 | 184 | 19 | 0.65 | 1.34 | 3.85 | 8.71 |
| 915 | F | 157 | 65 | 86 | 40 | 0.76 | 1.21 | 4.08 | 9.13 |
| 916 | F | 170 | 91 | 118 | 28 | 0.77 | 1.13 | 2.76 | 7.85 |
| 917 | F | 159 | 100 | 185 | 36 | 0.54 | 1.09 | 3.45 | 7.52 |
| 918 | F | 161 | 71 | 96 | 17 | 0.74 | 1.82 | 6.68 | 10.63 |
| 919 | F | 153 | 51 | 63 | 11 | 0.81 | 2.03 | 9.26 | 14.47 |
| 16 | M | 137 | 27 | 118 | 16 | 0.23 | 0.34 | 0.78 | 1.97 |
| 17 | F | 150 | 106 | 162 | 28 | 0.65 | 0.96 | 2.63 | 6.2 |
| 3065^5^  3064^5^ | M  M | 111  130 | 2  16 | 3  23 | 0  6 | 0.67  0.70 | 0.0  0.31 | -  1.25 | -  2.5 |
| 3063 | M | 100 | 3 | 4 | 2 | 0.75 | 0.0 | - | - |
| 921 | M | 168 | 22 | 22 | 22 | 1.0 | 1.5 | 2.68 | 3.0 |
| 2091 | M | 170 | 3 | 15 | 1 | 0.2 | 4.0 | 4.0 | 4.0 |
| 3 | M | 250 | 63 | 66 | 51 | 0.96 | 2.40 | 4.21 | 5.93 |
| 3066 | F | 119 | 5 | 14 | 2 | 0.36 | 0.0 | 0.0 | 0.0 |
| 3067 | M | 128 | 15 | 24 | 11 | 0.62 | 1.37 | 1.4 | 0.0 |
| 922 | M | 116 | 21 | 27 | 10 | 0.78 | 0.69 | 2.34 | 2.65 |
| 924 | M | 201 | 5 | 5 | 5 | 1.0 | 2.08 | 2.98 | - |
| Mean |  |  | 58 | 80 | 26 | 0.80 | 1.42 | 3.56 | 5.43 |

Transmitter numbers with identical numbered superscripts indicate individuals that were recaptured and fitted with an additional transmitter at a later date. Size, detection and activity space data reflect the two periods of monitoring for these individuals. STL, stretch total length; t_det_, number of days detected; t_max_, number of days from first to last detection; t_con_, maximum number of consecutive days present; RI, residence index; AS_d_, mean daily activity space; AS_w_, mean weekly activity space; AS_m_, mean monthly activity space;
